# Short-acting bronchodilators for the management of acute exacerbations of chronic obstructive pulmonary disease in the hospital setting: systematic review

**DOI:** 10.1186/s13643-018-0860-0

**Published:** 2018-11-29

**Authors:** Zoe A. Kopsaftis, Nur S. Sulaiman, Oliver D. Mountain, Kristin V. Carson-Chahhoud, Paddy A. Phillips, Brian J. Smith

**Affiliations:** 1 Faculty of Health Science, Division of Medicine, The University of Adelaide, Adelaide, South Australia Australia; 2 Clinical Practice Unit, The Queen Elizabeth Hospital, Adelaide, South Australia Australia; 3 Respiratory Medicine Unit, The Queen Elizabeth Hospital, Central Adelaide Local Health Network, Adelaide, South Australia Australia; 4 School of Health Sciences, University of South Australia, Adelaide, South Australia Australia; 5 Department of Medicine, Flinders University, Adelaide, South Australia Australia; 6 SA Health, Government of South Australia, Adelaide, South Australia Australia

**Keywords:** Chronic obstructive pulmonary disease, Short-acting bronchodilators, SABA, SAMA, Systematic review, Hospital, Exacerbation, Dose, Delivery

## Abstract

**Background:**

Currently, there is a lack of guidelines for the use of short-acting bronchodilators (SABD) in people admitted to hospital for acute exacerbation of chronic obstructive pulmonary disease (AECOPD), despite routine use in practice and risk of cardiac adverse events.

**Aim:**

To review the evidence that underpins use and optimal dose, in terms of risk versus benefit, of SABD for inpatient management of AECOPD and collate the results for future guidelines.

**Methods:**

Medline, Embase, the Cochrane Central Register of Controlled Trials, clinicaltrials.gov and International Clinical Trials Registry Platform were searched (inception to November 2017) for published and ongoing studies. Included studies were randomised controlled trials or controlled clinical trials investigating the effect of SABD (β2-agonist and/or ipratropium) on inpatients with a diagnosis of AECOPD. This review was undertaken in accordance with PRISMA guidelines and a pre-defined protocol. Due to heterogeneous methodologies, meta-analysis was not possible so the results were synthesised qualitatively.

**Results:**

Of 1378 studies identified, 10 met inclusion criteria. Narrative synthesis of 10 studies revealed no significant differences in most outcomes of interest relative to dose, delivery via inhaler or nebuliser, and type of β2-agonist used. However, some evidence demonstrated significantly increased cardiac side effects with increased dosage of β2-agonist (45% versus 24%), *P*<0.05).

**Conclusion:**

This review identified a paucity of methodologically rigorous evidence evaluating use of SABD among AECOPD. The available evidence did not identify any additional benefits for participants receiving higher doses of short-acting β2-agonists compared to lower doses, or based on type of delivery method or β2-agonists used. However, there was a small increase in some adverse events for participants using higher doses of β2-agonists.

**Electronic supplementary material:**

The online version of this article (10.1186/s13643-018-0860-0) contains supplementary material, which is available to authorized users.

## Introduction

The high prevalence of cardiac comorbidity and cardiac death in people with chronic obstructive pulmonary disease (COPD) [1, 2] may be exacerbated by the adrenergic effects of the routine frequent use of short-acting bronchodilators in clinical practice [3]. As a result, current guideline recommendations are to avoid prolonged use of β2-agonists [4]. Hence, there is a need to synthesise existing evidence for its use given that these agents are a mainstay of COPD exacerbation management, with some consideration of the risk/benefit, and optimal dosing.

Periods of acute exacerbation of COPD (AECOPD) often result in hospitalisation, wherein patients are at increased risk of decreased quality of life and death [5–8]. People suffering AECOPD experience an abnormal deterioration of respiratory symptoms defined as increased dyspnoea, sputum production and/or sputum purulence which may be infective or non-infective in origin [7].

The severity of an exacerbation is, in part, determined by the degree of dyspnoea experienced by the patient, largely driven by bronchoconstriction [9]. It is a universally accepted practice to administer rescue doses of short-acting β2-agonists and/or ipratropium known collectively as short-acting bronchodilators (SABD) to reduce this bronchoconstriction in AECOPD [9, 10].

However, cardiac markers, N-terminal prohormone of brain natriuretic peptide (NT-proBNP) and troponin T, are noted to be elevated in people suffering AECOPD and predict 30-day mortality independently of disease severity and prognosis [11]. Furthermore, the high prevalence of cardiovascular disease in people with COPD is well documented; despite this, the common comorbidity remains largely underdiagnosed [1, 2, 12–19]. Hence, unregulated, frequent administration of SABD in the hospital setting is concerning, given the adrenergic and pro-arrhythmic effects. There is some evidence of positive correlation between levels of short-acting β2-agonists and increased cardiac stress markers, specifically NT-proBNP [20].

Despite routine administration of SABD in the inpatient setting and the potential risk, there is no clearly evidence-based, standardised guideline for their use, particularly in terms of optimal and safe dosage [21]. Differences in clinical practice can be extrapolated down from geographic variances, between hospital differences and the dissimilarities in practice between individual physicians. Consultation of major international COPD guidelines provides little elucidation, with recommendations for SABD being underpinned by weak evidence, some of which has been extrapolated from studies of asthma patients and stable COPD [22, 23]. Overall, the evidence supporting the use of SABD for inpatients is unspecified to the inpatient setting [7, 22, 23]. Given that people with COPD are admitted to hospital frequently [24–26], optimisation and standardisation of COPD management, which includes SABD administration, is important to address large variability identified in current treatment [27, 28].

This systematic review was undertaken in order to evaluate the existing literature relating to SABD use for the inpatient management of AECOPD. Subsequently, these findings may be used as the foundation for clinical practice guidelines regarding specifications for the inpatient administration of SABD, including the use of one or a combination of agents, optimal and safe dose, most effective delivery method and duration of treatment.

## Methods

This systematic review was undertaken in accordance with the guiding principles of the Preferred Reporting Items for Systematic Reviews and Meta-Analyses (PRISMA) statement (see Additional file 1) [29]. Prior to commencing the review, a protocol was defined by the authors. This was not registered online in Prospective Register of Systematic Reviews (PROSPERO), and therefore, the review protocol will be described in detail in this section.

### Eligibility criteria

Studies were eligible for inclusion if they were randomised controlled trials or controlled clinical trials investigating the effect of SABD (short-acting β2-agonist and/or ipratropium) when administered to adult inpatients with a diagnosis of AECOPD. The comparison group could be either placebo, usual care, delayed intervention or another SABD (agent, dose or delivery method). Studies were included if the SABD was part of a wider treatment regime. The protocol stipulated that a study may be included if a maximum of 25% of the participants had a primary diagnosis other than COPD (e.g. participant is asthmatic or has secondary diagnosis of AECOPD) and if the data for COPD could be isolated. This parameter was implemented in acknowledgement of the high prevalence of comorbid conditions in patients with COPD [30–32]. Full-text and conference abstracts were eligible for inclusion where relevant outcomes were reported. In the case of abstracts with no published full text, authors were contacted in an effort to access more complete data for review.

### Outcomes

The authors selected both primary and secondary outcomes which were deemed to be the most clinically relevant to the topic. The primary outcome was selected, according to the clinical expertise of the clinician-authors, as the primary consideration for use of SABD to treat AECOPD in the hospital setting.

The primary outcome in this review was treatment failure. For the purposes of this review, this was defined as (1) mortality as a result of AECOPD, (2) intensive care unit/critical care unit (ICU/CCU) admission or (3) respiratory failure requiring non-invasive or mechanical ventilation. Secondary outcomes included spirometric values, i.e. forced expiratory volume in 1 s (FEV^1^) and forced vital capacity (FVC), dyspnoea, sputum production/purulence and quality of life. Other outcomes of interest were hospital utilisation (length of stay, hospital admission/readmission rate, emergency department presentations) and adverse events related to β2-agonist and anticholinergic agents (including cardiovascular events).

### Search strategy

A keyword search strategy for systematic literature searching was devised and refined after a preliminary search by ZK and NS in consultation with a senior Reference Librarian, with the goal of identifying all relevant studies (see example in Additional file 2). Both keywords and MeSH terms were used where appropriate; hence, a combination of the following was used: COPD, inpatient, hospital and bronchodilator agents; all variants of these terms were also included. Electronic databases searched (all years up to 2017) for published literature were Medline (OVID), Embase (OVID) and the Cochrane Central Register of Controlled Trials (CENTRAL). Grey literature was searched online via ClinicalTrials.gov and the International Clinical Trials Registry Platform to identify relevant ongoing studies. Supplementing this, alerts were set for the searched databases to ensure any recently published studies were identified prior to publishing this review. Screened studies were not restricted by language or date. Bibliographies of included articles and reviews were hand searched for identification of any other studies potentially eligible for inclusion. Online searches are current as of November 2017.

### Data extraction

Screening was undertaken by two independent reviewers. First, titles and abstracts of identified citations were appraised, after duplicates were removed. Following this, full text was obtained for the remaining studies and compared to the pre-established inclusion criteria, before final inclusion in the review. Data pertaining to the above-mentioned primary and secondary outcomes as well as participant and study design characteristics were extracted into a standardised, pilot-tested form (see Additional file 3). This was done independently by a combination of two authors (ZK and either NS or OM). If any disagreements had occurred during this process that could not be resolved by consensus, a third member of the author team would have been consulted.

### Methodological quality

Consensus was reached regarding methodological quality of the included studies after independently evaluating each of them in keeping with the Cochrane Collaboration’s tool for assessing risk of bias [33]. Using this method, each study was rated as having low, unclear or high risk of bias for domains including “selection bias”, which is split into two sub-groups relating to the appropriateness of the randomisation procedure and allocation of participants to study groups. Presence of “performance bias” and “detection bias” relating to the blinding of participants, personnel and outcome assessment was also assessed, in addition to “attrition bias” and “reporting bias” which relate to incomplete outcome data and selective reporting. The domain “other bias” relates to any potential sources of bias which are not covered by the aforementioned categories, for example, contamination between intervention and control groups or bias arising from particular study designs (e.g. lack of washout period in a cross-over trial).

### Data analysis

Extracted data was synthesised descriptively based on published data only. The data presented in the included studies were reviewed critically by the authors, taking into account the outcome of the risk of bias assessment when synthesising the evidence. Meta-analysis would have been undertaken according to the predefined protocol, if two or more studies were sufficiently similar in methodology to result in a meaningful outcome. However, included studies used heterogeneous methods specifically relating to the measurement of outcomes and intervention delivery, and hence, a meaningful quantitative analysis was not possible in this instance.

## Results

**Fig. 1:**
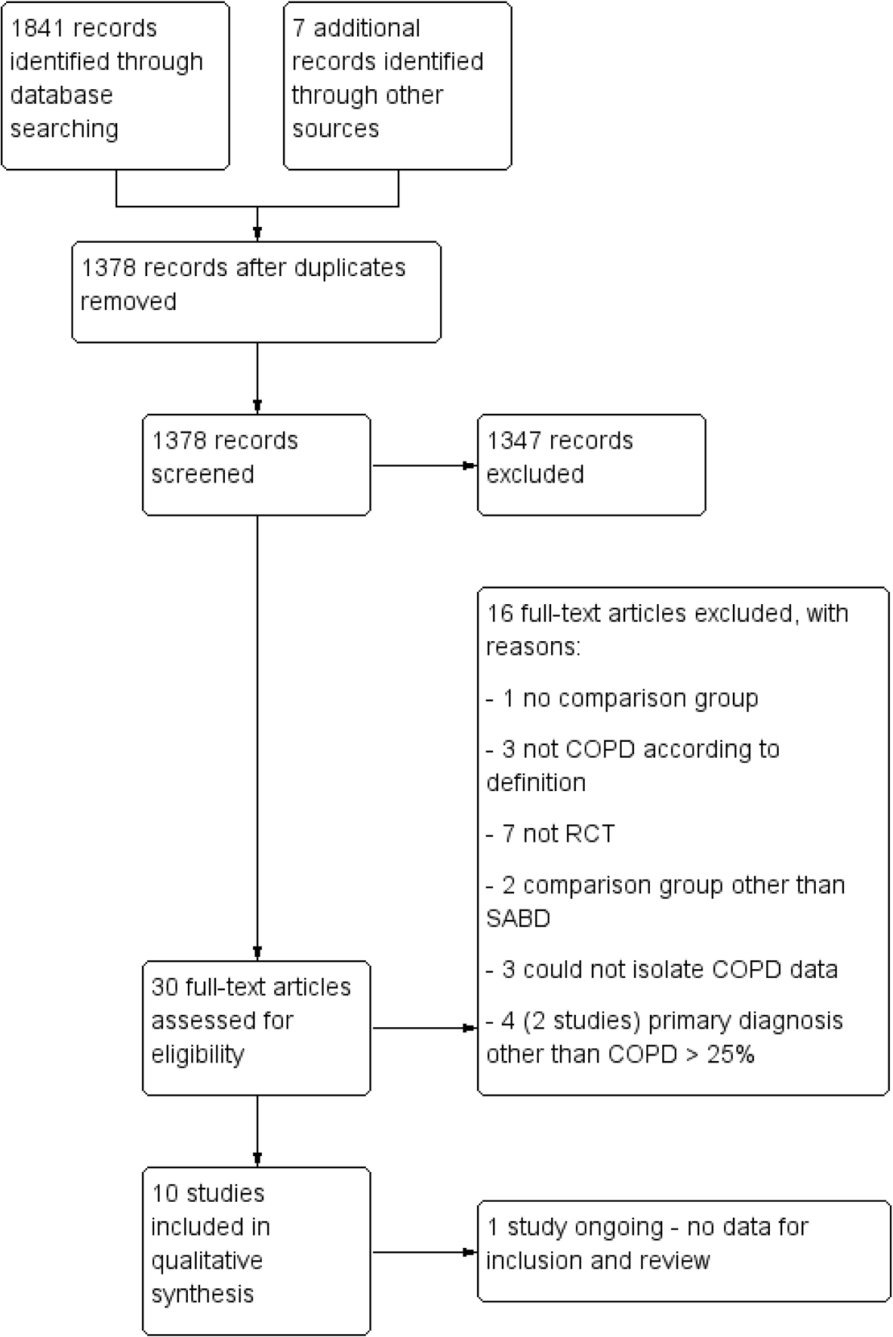
PRISMA study flow diagram of included studies

The systematic literature search returned 1848 citations; after de-duplication, 1378 studies were screened, and 10 met all criteria for inclusion in this review (Fig. 1). One study was identified via a search of online clinical trials registries and was deemed to be relevant for inclusion; however, it is still ongoing and no data were available for this review [34]. Included studies originated from six different countries and were dated from 1989 to 2016. Of these, two studies were available in abstract form only despite attempts to contact the original authors for further details [35, 36]. Characteristics of the included studies can be seen in Table 1. Overall, the pool of included studies comprised of eight RCTs [35–42], a randomised crossover trial [43] and a controlled clinical trial [44]. Study participants were people with COPD with average age above 60 years for all.

**Table 1:** Characteristics of included studies

Study				Participants			Intervention		
	Setting	Design	Sample size	Baseline FEV1 mean (SD)	Age, years mean (SD)	Gender	Group 1	Group 2	Outcomes
Berry [43]	USA, single centre	Double blind RXT	20	0.69 ± 0.28 L	67.9 ± 7.1	20M	MDI + spacer placebo 4 puffs and nebulized albuterol 2.5 mg over 10–15 min	MDI + spacer albuterol 4 puffs of 0.36 mg and nebulized placebo	FEV^1^ FVC Dyspnoea (Borg) Adverse events
Cromheecke [44]	The Nether-lands, single centre	CCT	42	Group 1 47.39 ± 2.94% Group 2 50.48 ± 3.75%	Group 1 71.1 ± 1.9 Group 2 71.7 ± 1.6	26M 16F	Nebulized salbutamol 10 mg	Nebulized salbutamol 5 mg	FEV^1^ FVC Adverse events Dyspnoea (Borg)
Cushen [36]	Ireland, single centre	RCT	31	48 ± 18%	NR	NR	Combined salbutamol 2.5 mg/ipratropium bromide 0.5 mg via vibrating mesh nebuliser	Combined salbutamol 2.5 mg/ipratropium bromide 0.5 mg via jet nebuliser	FEV^1^ FVC Dyspnoea (Borg)
Emerman [37]	USA, single centre	RCT	86	Group 1 24.5 ± 11.7% Group 2 24.9 ± 11.2%	63.9 ± 8.9	42M 44F	Albuterol 2.5 mg MDI every 60 min (total 2) with saline MDI at other treatment times (total 4)	Albuterol 2.5 mg MDI every 20 min (total 6 doses)	FEV^1^ FVC Hospital utilisation Adverse events
Formgren [38]	Sweden, single centre	Double blind RXT	15	35.6% (SD NR)	61 ± 9	11M 4F	Placebo turbuhaler + placebo MDI	1) Terbutaline 1 mg via turbuhaler + placebo MDI 2) Terbutaline 1 mg via MDI + placebo turbuhaler 3) Terbutaline 2.5 mg via turbuhaler + placebo MDI with spacer 4) Terbutaline 2.5 mg via MDI with spacer + placebo turbuhaler	FEV^1^ FVC
Moayyedi [39]	UK, single centre	RCT	62	Group 1 0.77 ± 0.34 L Group 2 0.78 ± .41 L	Group 1 70.4 ± 9.1 Group 2 67.8 ± 6.7	NR	5 mg nebulised salbutamol 4 times per day + standard of care	5 mg nebulised salbutamol + 500 μg ipratropium bromide 4 times per day + standard of care	FEV^1^ FVC Dyspnoea (subjective) Hospital utilisation
Nair [40]	UK, multicentre	Double-blind RCT	86	0.82 ± 0.41 L	69.3 ± 9.3	39M 47F	2.5 mg nebulized albuterol 4 hourly	5 mg nebulized albuterol 4 hourly	FEV^1^ Hospital utilisation Adverse events
Shortall [41]	USA, multicentre	Non-blinded RCT	34	0.75 L (0.5–2.02 L)	Group 1 67 Group 2 71	25M 9F	40 mg oral methylprednisolone 6 hourly until asymptomatic then 40 mg daily + *MDI albuterol (maximum of 20 puffs 4 hourly) and ipratropium bromide (maximum 8 puffs 4 hourly)* + cefuroxime 500 mg twice daily	40 mg IV methylprednisolone every 6 h until asymptomatic then 40 mg oral daily + *2.5 mg nebulized albuterol and 0.5 mg ipratropium bromide 4 hourly* + cefuroxime 1.5 g IV 8 hourly for 36 h then 500 mg orally twice daily	FEV^1^ Hospital utilisation Treatment failure Dyspnoea (Borg) Adverse events
Willaert [42]	Belgium, single centre	RCT	48	Group 1 0.70 ± 0.27 L Group 2 0.82 ± 0.46 L	Group 1 71 ± 8 Group 2 72 ± 6	42M 6F	32 mg oral methylprednisolone for 7 days before tapering off + *1.6 mg fenoterol and 640 μg ipratropium bromide daily via MDI + spacer*	40 mg IV methylprednisolone daily for 10 days before tapering off + *10 mg salbutamol and 1 mg ipratropium bromide aerosolised daily*	FEV^1^ FVC Treatment failure Hospital utilisation Dyspnoea (subjective) Quality of life (CRQ)
Wollak [35]	USA, single centre	RCT	15	NR	NR	NR	2.5 mg nebulized albuterol 4 hourly + standard of care	5 mg/h continuous nebulized albuterol for 4 h, then 2.5 mg intermittently every 4 h + standard of care	FEV^1^ FVC

Key: *SD* standard deviation, *USA* United States of America, *RXT* randomised crossover trial, *M* male, *MDI* metred dose inhaler, *FEV*^*1*^ forced expiratory volume in 1 s, *FVC* forced vital capacity, *CCT* controlled clinical trial, *F* female, *RCT* randomised controlled trial, *NR* not reported, *UK* United Kingdom, *CRQ* chronic respiratory questionnaire

### Risk of bias assessment

**Fig. 2:**
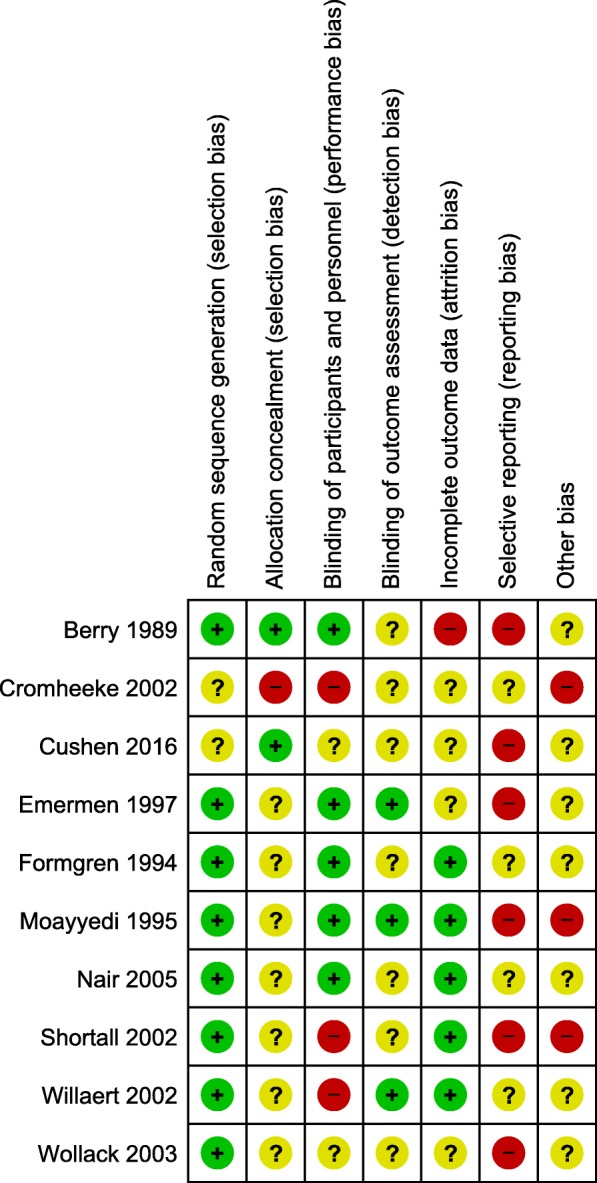
Risk of bias assessment for individual included studies

**Fig. 3:**
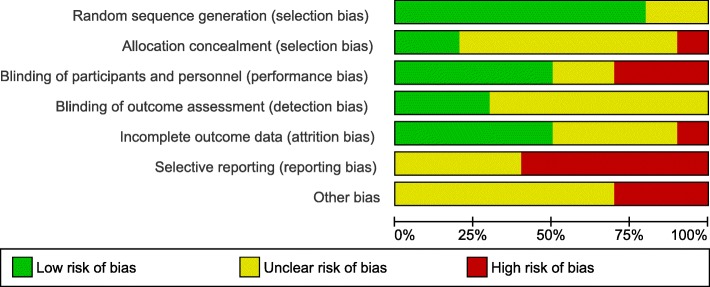
Risk of bias summary for included studies

The overall quality of the included studies was assessed as average to poor, despite the majority of studies being randomised controlled trials. This was mainly due to inadequate reporting of methodology, which gave many studies an unclear risk of bias. There was a low risk of bias with regard to the randomisation and sequence generation. When it was specified, participants were usually randomised in 1:1 ratio blocks and one study randomised using the Latin square design. One study [44] had a high risk of bias for this domain as there was a change to protocol during the study, such that randomisation was not possible. Blinding was performed variably among the studies, with many studies having incomplete blinding or open label testing. Studies where only the abstract was available were particularly susceptible to incomplete or brief descriptions of methodology. Risk of bias for individual studies is presented in Fig. 2, and a summary of risk of bias is presented in Fig. 3.

Included studies were of small sample size (range *n* = 15 to *n* = 86), and justification for sample size and relevant power calculations were not reported in the publications across the board.

### Treatment failure

Although treatment failure was the primary outcome of this review, only two studies [41, 42] reported it, with heterogeneous research methodologies; thus, meta-analysis was not possible. One of these studies, with 50 participants, demonstrated a similar rate of treatment failure with oral/metred dose inhaler (MDI) (32%) and intravenous (IV)/nebuliser (33%) (*P* = 1.0) [41]. However, these treatment failures were considered to be minor (defined as a subjective perception of clinical deterioration by either participant or physician) and did not satisfy the definition stated in the protocol of this review. The other study [42] noted incidences of treatment failure as defined by our criteria; however, these participants were withdrawn from the original sample of 48 and excluded from analysis. Despite this, the authors reported treatment failure as one of their outcomes in their description of methods. In the case of different treatment regimes, IV/aerosol therapy resulted greater ICU admissions compared to oral/MDI (3 vs 1) [42].

### Forced expiratory volume in 1 s

Of the 10 included studies which reported FEV^1^ as an outcome, nine reported that all groups experienced an increase from baseline with administration of SABD [36–44]; the improvement from baseline could not be determined from one of the included abstracts [35]. None of the studies reviewed showed a significant difference between groups for FEV^1^ regardless of variances in types of SABD, delivery method or dose [35, 40–44]. However, for one study (*n* = 42), authors reported a non-significant average increase in FEV^1^ of 11.2% with 10 mg of salbutamol versus 6.9% with 5 mg [44]. Another study, with 86 participants, demonstrated higher percentage increase in FEV^1^ at 120 min compared to baseline with a higher dose regimen of albuterol (29.2 ± 35.9 vs 15.1 ± 36.2; *P* = 0.09) though again this was non-significant [37]. From one small RCT (*n* = 20), use of a nebuliser appeared to improve the percent change in FEV^1^ at 1 h post-treatment compared to MDI plus spacer (16.7% ± 17.0 vs 13.4% ± 20.5). However, authors reported that this was a non-significant and both groups’ improvements appear to be clinically relevant compared to pre-treatment values [43].

### Forced vital capacity

Of the 10 included studies, seven reported FVC as an outcome, all of which reported an improvement from baseline regardless of group.

Four studies reported a statistically significant difference between intervention and control groups. Salbutamol at 10 mg compared to 5 mg was reported to have significantly improved FVC at 10 min post-administration; although the exact *p* value was not reported for this time point, the magnitude of difference between the two groups was approximately 11.5% [44]. However, after 10 min, authors reported the statistical significance of the between groups difference was not sustained for subsequent time points up to 240 min [44]. Other studies investigating different dosing approaches did not report any evidence of an effect on FVC with a higher versus lower dose [37, 40]. Both of these studies had a sample size of 86 and were the two largest studies included for review.

In a study of 62 people with COPD, monotherapy (salbutamol 5 mg) alone compared to combination therapy (salbutamol 5 mg plus ipratropium 500 μg) demonstrated a significantly higher mean change in FVC at 3 days post-administration (0.25 L (0.42) vs 0.04 L (0.41), 95% CI − 0.42 to 0.001; *P* = 0.05); this difference was no longer significant on days 7, 14 and discharge [39].

In terms of delivery method, continuously nebulised albuterol (− 0.85% ± 73.38) appeared inferior to intermittently nebulised albuterol (21.22% ± 30.73) for percentage change in FVC at 4 h, *P* = 0.043, reported as absolute change in FVC [35]. Nebulised albuterol resulted in a higher mean change in FVC 1 h after treatment (310 mL ± 370) compared to albuterol via MDI plus spacer (190 ± 360); however, this difference was not significant [43]. Furthermore, vibrating mesh nebuliser (salbutamol 2.5 mg plus ipratropium 0.5 mg) demonstrated a greater increase in FVC (0.40 L ± 0.39) compared to standard hospital jet nebuliser (same drug regime) (0.19 L ± 0.19); however, this was not significant, *P* = 0.06. A subsequent study reported that the addition of a spacer and/or an increase in dose from 1.0 to 2.5 mg (terbutaline) did not confer any additional benefit to FVC [38]. A differing treatment regime including nebulised salbutamol plus ipratropium compared to fenoterol plus ipratropium via MDI with spacer also failed to demonstrate a difference between the groups [42].

### Dyspnoea

Dyspnoea was reported in six of the included studies: four utilising the Borg scale [36, 41, 43, 44] while the remaining two studies [39, 42] used subjective description including visual analogue scale. All six studies demonstrated improvement in dyspnoea post-intervention compared to baseline. The only study investigating differing doses found no difference between administration of 5 mg or 10 mg of salbutamol [44].

Similarly, the two studies that investigated oral/MDI versus IV/nebuliser showed no significant differences between groups with *P* = 0.75 [41] and *P* = 0.15 [42] respectively. There was a greater change in dyspnoea measured by Borg scale when MDI plus spacer albuterol was administered (− 1.08 ± 2.01) compared to nebulised albuterol (− 0.73 ± 1.75), but this did not reach statistical significance [43]. Another study comparing two different types of nebulisers (vibrating mesh versus standard hospitalised jet) showed no statistical difference between group comparison [36]. The addition of ipratropium to nebulised salbutamol was not found to be more effective in reducing self-reported dyspnoea than nebulised salbutamol alone [39].

### Sputum production/purulence

None of the included studies evaluated effect of SABD on sputum production or purulence.

### Quality of life

Only one study reported on quality of life as an outcome. Using the chronic respiratory disease index questionnaire [42], the study did not demonstrate a significant difference when comparing oral/MDI versus IV/nebuliser (86 ± 20 vs 90 ± 24, *P* = 0.73) for this outcome. However, both groups improved in quality of life from baseline regardless of intervention; the IV/nebuliser group by approximately 20% (*P* = 0.036) and the oral/MDI group by approximately 45% (*P* = 0.0069).

### Adverse events

Five of the included studies reported adverse events relating to β2-agonists including changes to heart rate [40, 41, 43, 44], palpitations [40], tremor [40, 44] and changes to blood pressure [43, 44]; one study reported on albuterol-related side effects as a general outcome without further qualification [37]. One study found no difference between 2.5 mg and 5 mg of albuterol [40] with regard to heart rate and palpitations, but tremor was common with the higher dose (5 mg). This study reported overall adverse events were similar in both groups (*P* = 0.506) [40]. Another study comparing 10 mg salbutamol versus 5 mg salbutamol demonstrated higher increase in heart rate with 10 mg salbutamol dose at 30 min post-administration (76.9 ± 4.3 versus 74.7 ± 3.27 bpm). While this was reported as significant, there was no supporting *p* value [44]. Salbutamol 10 mg led to an earlier maximal change in heart rate (*t* = 30 min) compared to salbutamol 5 mg (*t* = 120 min). Furthermore, there was a significant decrease in systolic blood pressure in the 10 mg salbutamol at *t* = 10 min (10.2 ± 13.7 mmHg). Similarly, diastolic blood pressure was also significantly lower with 10 mg salbutamol compared to baseline, reaching nadir *t* = 30 min; 5 mg salbutamol also significantly decreased from baseline, but the reduction occurred later at *t* = 60 min and the minimum was smaller (5.8 ± 2.0 mmHg versus 10.2 ± 1.7 mmHg). Area under curve for tremor was greater in the 10 mg salbutamol (1139.9 ± 95.5) compared to 5 mg salbutamol (892.4 ± 61.0), *p* value not reported [44]. A study looking at cumulative dose of 15 mg versus 10 mg albuterol showed significantly higher adverse events in the higher cumulative dose (45% versus 24%) (*P* < 0.05). However, this study did not specify the type of adverse events other than to say “side effects consistent with albuterol treatment” [37].

There was no difference in heart rate (2.7 ± 9.0 bpm versus 2.9 ± 7.4 bpm) with administration of SABD via MDI plus spacer or nebuliser [43]. Similarly, there was no difference in systolic/diastolic blood pressure 5 min post-treatment with MDI plus spacer (2.7 ± 11.8/2.5 ± 12.5 mmHg) compared to nebuliser (0.8 ± 15.2/3.7 ± 13.2 mmHg) [43].

In a single study where participants received therapy via either oral/MDI or IV/nebuliser regime (*n* = 50), there was no significant difference reported between groups in terms of heartrate post-treatment [41]. Any differences noted by the authors, from baseline, were consistent with an expected physiological response to SABD administration (i.e. increase < 10 bpm) [41].

The only study reporting use of ipratropium did not report adverse events [39].

### Hospital utilisation

Six of 10 studies investigated hospital utilisation as an outcome [37, 39–43].

The study comparing cumulative higher dose regime to lower overall dose resulted in equal rates of hospitalisation (69% for both groups) [37]. The addition of ipratropium to salbutamol did not improve the hospital length of stay in one study [39]. However, it appears administering an oral/MDI SABD treatment regime compared to an IV/nebuliser regimen may have some (though non-significant) reductive effect on length of stay (10.6 ± 2.8 vs 15.5 ± 10.3, *P* = 0.06) [42].

Dosing with 2.5 mg versus 5 mg of albuterol produced a reduced length of stay (6 vs 9 days) though this was not statistically significant (*P* = 0.084) [40]. Adding ipratropium to salbutamol resulted in additional, though non-significant, mean length of stay: 11.8 ± 4.4 vs 10.5 ± 4.7 days (95% CI − 1.02 to 3.62) (*P* > 0.05) [39]. Two studies demonstrated a reduced but non-statistically significant length of stay with an oral/MDI with spacer treatment regime (antibiotics, steroids and SABD) compared to IV/nebuliser 11 ± 3 versus 16 ± 10 days (*P* = 0.06) [42] and 4.3 vs 5.1 days (*P* < 0.56) [41].

A 5-mg vs 2.5-mg regimen of albuterol resulted in higher cumulative cost £8665 versus £4048 (£188.37 vs £101.20 per patient) [40].

Only one study reported hospital readmissions as an outcome, following up participants 20 weeks post-discharge [42]. At this time, 42% of the 23 participants (approximately 10 individuals) in the IV/nebuliser treatment group experienced a readmission versus 66% of 25 participants (17 individuals) in the oral/MDI with spacer treatment group.

## Discussion

Established guidelines for the management of COPD exacerbations recommend the use of SABD [7, 23]. The effectiveness of SABD in stable COPD is well established [45, 46], and it is this evidence that has been generalised for use in the inpatient setting [47–50]. However, inpatient administration of SABD is underpinned by weak evidence and is not specific to patients with AECOPD [7, 22, 23]. As such, this review aimed to evaluate the existing evidence for the effectiveness of SABD for patients admitted to hospital due to AECOPD. A paucity of data was identified with only 10 studies meeting the eligibility criteria for inclusion, the majority of which had small sample size, poor methodological quality and/or lacked adequate reporting of the study design. This is likely a reflection of looser reporting standards for older studies (only one included study was published after 2005). Further, there appears to be a lack of interest within the clinical and research space toward focussing new endeavours and funding on what is widely considered a well understood, mainstream treatment for AECOPD. However, as evidenced by the findings of this review, there are still gains to be made particularly for outcomes relating to hospital utilisation, spending and a baseline for standardisation of care. The prevalence of COPD and subsequent high levels of hospitalisations warrant a renewed interest in this fundamental area of inpatient management of these patients [51]. Through evaluation of patient and hospital-based outcomes, we attempted to determine the optimal dose, delivery method, duration and types of agents for the hospital setting, as described in the proceeding sections.

### Dose evaluation

Comparison of dosages of SABD for inpatient application demonstrated no added clinical bronchodilatory benefit with doses above 2.5 mg of salbutamol per administration [35, 40, 44]. In fact, there was some indication that a higher dose may result in a higher number of adverse events, with hypotensive and arrhythmic effects noted with > 10 mg of salbutamol [44]. This finding is consistent with a recent study demonstrating a positive correlation between blood salbutamol levels and N-terminal prohormone of brain natriuretic peptide in people treated with rescue relief for AECOPD [20]. Furthermore, this review indicates that with 5 mg, there was a trend towards increased incidence of tremors, though this was not significant [40]. The evidence suggests that using a lower dose of SABD (e.g. salbutamol 2.5 mg) is just as effective as a higher dose (e.g. 5 mg) in achieving relief of symptoms of breathlessness associated with AECOPD and may prevent unnecessary risk to patients due to drug-related adverse events. Moreover, the use of less medication may potentially result in cost savings with reduced dose delivery per patient for the same clinical benefit [40].

Similarly, the sole study looking at terbutaline did not confer additional benefit beyond dose of 1 mg compared to 2.5 mg [38].

### Delivery method

Of the five studies [36, 38, 41–43] reporting different delivery methods for SABD, two were embedded into a wider treatment regime [41, 42]. Both of these studies used a similar approach, differing mainly in the use of either IV or oral steroids [42] or IV/oral antibiotics [41]. The combination of IV steroid with nebulised SABD is likely a model for treating a high-dependency patient requiring more support than a patient who can tolerate an oral steroid and navigate an MDI with spacer [42]. While consistent use of either oral or IV steroid would have been preferable in methodological terms for clarity of comparison, unpacking the results of this study with reference to other research offers interesting and practical insights into clinical decision-making across the diverse spectrum of patients. A systematic review concluded that in the treatment of AECOPD, there is no discernible difference between treating with and IV or oral steroid approach [52]. Furthermore, a recent Cochrane Review concluded, much like the results of this review, that while there is a dearth of large well-conducted studies in this area, there does not appear to be a difference between treating with nebulised or MDI with spacer SABD when considering FEV^1^ [53]. The lack of conclusive results in favour of one or the other suggests that clinicians can feel comfortable using which ever delivery method best suits the needs and capabilities of their individual inpatients. One study investigated MDI with spacer versus nebulised SABD with both groups receiving equivalent standard therapy which included use of IV aminophylline and corticosteroids and additional oral or inhaled sympathomimetics [43]. The standard therapy outlined in the latter study is now considered outdated and no longer considered standard practice.

Overall, despite variations in methodology, all five studies demonstrated no significant difference between delivery of SABD via either nebuliser or MDI with spacer. In this instance, clinicians may be guided more by the patient presentation and tolerability when determining which device to use, rather than potential efficacy of the device.

### Duration of treatment

No studies investigated the optimal time frame for duration of SABD administration before termination in the inpatient setting for AECOPD. This is an important aspect of the treatment regime and requires further investigation.

### Mono- versus co-intervention therapy

All studies showed an improvement from baseline with administration of SABD; this is not surprising given established evidence of effectiveness in stable COPD [46]. There appeared to be no difference between mono- and co-intervention therapy with regard to FEV^1^. As a result of these findings, it is possible to suggest that monotherapy with a β2-agonist for inpatients with AECOPD to be sufficient and addition of anticholinergic agents is of no additional clinical benefit and a potential waste of resources. However, this is based on a single included study with only 62 participants, and as such, results should be interpreted with caution. Further research in this area is required prior to considering changes to practice.

### Type of short-acting β2-agonist

Most included studies reported the use of either salbutamol or albuterol which are the same agent [35, 39–41, 43, 44]. One study investigated a treatment regime which delivered albuterol to one group and fenoterol to the other group [42]; while both are short-acting β2-agonists, the latter has a slightly longer duration of action [54]; another study examined terbutaline [38]. There appeared to be no difference between agents used. Interestingly, fenoterol has been withdrawn from the pharmaceutical market due to its association with increased risk of death [55]. Based upon this observation, a choice may be made according to clinical preference, safety profile and drug availability.

## Limitations of this review

The limitations of this review owe mainly to the quality and quantity of included studies. The majority of the included studies were published before 2008, bar one [36]. Hence, recommendations based on these findings may not be a true reflection of current clinical practice. This is perhaps an indication that clinical practice is evolving quickly, with research and the evidence-base lagging behind. Furthermore, the wider literature on this general topic is also somewhat lacking in recency; it appears that perhaps the profession has just accepted SABD as a relatively non-threatening and straight forward treatment for AECOPD and is happy to focus research efforts on other, more exciting things. This is of concern given that administration of SABD is considered a core therapy during AECOPD and standardisation of practice is desirable. The heterogeneity of reported methodologies and outcomes made meta-analysis of the data impossible. Furthermore, as indicated by the risk of bias assessment, the included studies were of average to poor methodological quality with small sample sizes, again limiting generalisability of the findings. It is also desirable to be able to assess the pooled effect of these studies to be able to provide a more objective assessment of effectiveness through meta-analysis; however, this was precluded in this review.

### Recommendations for clinical practice and future research

Given that a clear clinical benefit was not identified with use of higher doses of SABD, it is important to consider the risk of frequent and sustained use, while questioning continued acceptance of the status quo. A moderated approach is recommended, especially when administering four hourly nebulisations; extra care in the case of people with known or possible unknown comorbid cardiac disease is necessary as in these patients, risk may outweigh benefit.

Methodologically rigorous evaluations are required that evaluate the current use of SABD in the hospital setting following patient admission for AECOPD, particularly given the lack of recent evidence identified in this review. Future studies should report on all the outcomes pre-specified in this review, as these outcomes reflect not only patient level variables, but also those necessary for policy and guideline decision-making. Detailed description of dose, delivery method, duration and types of agents need to be described in detail to facilitate accurate evidence-based recommendations for the use of SABD in the inpatient setting.

## Conclusion

Despite the routine use of SABD in the hospital setting for the management of AECOPD, a paucity of data pertaining to the inpatient applications of this medication was found. Though the evidence is limited, it appears consideration of the use of a single short-acting β2-agonist such as salbutamol/albuterol at a dose of 2.5 mg may be sufficient to get the desired clinical outcome. Co-intervention therapy and higher dose regimens were not associated with any discernible clinical benefit for the outcomes reported in this review. Delivery method may be prescribed based on patient preference given the equivalence observed in delivery method for available data. However, despite the somewhat interesting results presented in this review, interpretation should be made with care as the quality of the evidence underpinning these outcomes were overall low.

The gap in evidence highlights the need for methodologically rigorous, appropriately powered studies to address the use of SABD specifically in the inpatient setting, to allow for improved patient- and hospital-based outcomes, and the basis of standardised practice with regard to prescription of mainline therapy of AECOPD.

## Additional files

Additional file 1: PRISMA checklist. (PDF 214 kb)

Additional file 2: Search strategy and search terms. (PDF 287 kb)

Additional file 3: Standardised data extraction template. (PDF 82 kb)

## Abbreviations

95%CI: 95% confidence interval
AECOPD: Acute exacerbation of chronic obstructive pulmonary disease
CENTRAL: Cochrane Central Register of Controlled Trials
COPD: Chronic obstructive pulmonary disease
FEV^1^: Forced expiratory volume in 1 s
FVC: Forced vital capacity
ICU/CCU: Intensive care unit/critical care unit
IV: Intravenous
MDI: Metered dose inhaler
NT-proBNP: N-terminal prohormone of brain natriuretic peptide
*P*: Probability value
PRISMA: Preferred Reporting Items for Systematic Reviews And Meta-Analyses
SABA: Short-acting β2-agonist
SABD: Short-acting bronchodilator
SAMA: Short-acting muscarinic agent
vs: Versus
