# Supplementary material for: Short-acting bronchodilators for the management of acute exacerbations of chronic obstructive pulmonary disease in the hospital setting: systematic review
Source: Syst Rev. 2018 Nov 29;7:213. doi: 10.1186/s13643-018-0860-0 (PMC6264607; doi:10.1186/s13643-018-0860-0)
Supplement: Supplementary file 2 — Search strategy and search terms. (PDF 287 kb) [file 13643_2018_860_MOESM2_ESM.pdf]

Search Strategy

Search strategy to identify included studies in MEDLINE:

1 Pulmonary Disease, Chronic Obstructive/
2 chronic obstructive pulmonary disease.mp. [mp=title, original title, abstract, mesh headings, heading words, keyword]
3 copd.mp. [mp=title, original title, abstract, mesh headings, heading words, keyword]
4 chronic obstructive lung disease.mp.
5 coad.mp. [mp=title, original title, abstract, mesh headings, heading words, keyword]
6 chronic obstructive airway disease.mp.
7 1 or 2 or 3 or 4 or 5 or 6
8 inpatients/
9 inpatient*.mp. [mp=title, original title, abstract, mesh headings, heading words, keyword]
10 exp hospitalization/
11 hospitalis*.mp. [mp=title, original title, abstract, mesh headings, heading words, keyword]
12 hospitaliz*.mp. [mp=title, original title, abstract, mesh headings, heading words, keyword]
13 (admission adj5 hospital).mp. [mp=title, original title, abstract, mesh headings, heading words, keyword]
14 8 or 9 or 10 or 11 or 12 or 13
15 exp Bronchodilator Agents/
16 bronchodilator*.mp. [mp=title, original title, abstract, mesh headings, heading words, keyword]
17 exp adrenergic beta-agonists/ or exp adrenergic beta-2 receptor agonists/
18 beta 2 agonist*.mp.
19 beta agonist*.mp.
20 adrenergic beta agonist*.mp. [mp=title, original title, abstract, mesh headings, heading words, keyword]
21 15 or 16 or 17 or 18 or 19 or 20
22 short acting.mp. [mp=title, original title, abstract, mesh headings, heading words, keyword]
23 21 and 22
24 saba.mp. [mp=title, original title, abstract, mesh headings, heading words, keyword]
25 albuterol.mp. [mp=title, original title, abstract, mesh headings, heading words, keyword]
26 exp Albuterol/
27 salbutamol.mp.
28 exp Terbutaline/
29 terbutaline.mp. [mp=title, original title, abstract, mesh headings, heading words, keyword]
30 Ipratropium/
31 ipratropium.mp. [mp=title, original title, abstract, mesh headings, heading words, keyword]
32 Isoproterenol/
33 isoproterenol.mp. [mp=title, original title, abstract, mesh headings, heading words, keyword]
34 bitolterol.mp.
35 Fenoterol/
36 fenoterol.mp. [mp=title, original title, abstract, mesh headings, heading words, keyword]
37 Isoetharine/
38 isoetharine.mp. [mp=title, original title, abstract, mesh headings, heading words, keyword]
39 levalbuterol.mp.
40 Metaproterenol/
41 metaproterenol.mp. [mp=title, original title, abstract, mesh headings, heading words, keyword]
42 pirbuterol.mp. [mp=title, original title, abstract, mesh headings, heading words, keyword]
43 Procaterol/
44 procaterol.mp. [mp=title, original title, abstract, mesh headings, heading words, keyword]
45 procaterol.mp. [mp=title, original title, abstract, mesh headings, heading words, keyword]
46 oxitropium.mp.
47 isoprenaline.mp.
48 isoetarine.mp. [mp=title, original title, abstract, mesh headings, heading words, keyword]
49 orciprenaline.mp.
50 24 or 25 or 26 or 27 or 28 or 29 or 30 or 31 or 32 or 33 or 34 or 35 or 36 or 37 or 38 or 39 or 40 or 41 or 42 or 43 or 44 or 45 or 46 or 47 or 48 or 49
51 23 or 50

52 7 and 14 and 51
