# Supplementary material for: Short-acting bronchodilators for the management of acute exacerbations of chronic obstructive pulmonary disease in the hospital setting: systematic review
Source: Syst Rev. 2018 Nov 29;7:213. doi: 10.1186/s13643-018-0860-0 (PMC6264607; doi:10.1186/s13643-018-0860-0)
Supplement: Supplementary file 3 — Standardised data extraction template. (PDF 82 kb) [file 13643_2018_860_MOESM3_ESM.pdf]

Short acting bronchodilators for the inpatient management of COPD.

Study quality assessment & data extraction form

Reviewers Name:

Date of form completion:

RevMan Study ID

References to this Trial

Check other references identified in searches. If there are further references to this trial (i.e. multiple publications for the same study) link the papers now & list below. All references to a trial should be linked under one *Study ID* in RevMan.

Code each paper	References<br>Format: [Surname] [initial], (etc). [Year of publication] [Title of paper]. [*Journal title*]; [volume] [(issue)]: [page numbers]

Methods:

	Further details
Country:	
Design: (e.g., RCT)	
Objective: (Aim of the study)	
Study Site: (Hospital, specialist sleep clinic, research institute etc.)	
Methods of analysis: (e.g., Logistic regression etc.)	
Adjustment for clustering	

Participants:

(If multiple intervention/control arms label as 1. 2. 3. etc.)	Further details<br>Intervention:	Control:
Eligible for study (n-value):		
Randomised: (n-value for each arm of study randomised)		
Completed: (participant completion n-value for each		

arm of study)		
Age: (mean value &/or range for each arm)		
Gender: (for each arm)		
COPD diagnosis criteria		
Recruitment: (through what means, e.g., outpatient clinics etc.)		
Diseases/co-morbidities included:		
Reasons for Subject Exclusion:		

Interventions:

	Further details
Setting:	
Duration of intervention: (weeks and hours)	
Intervention description	
Control description	

Outcomes:

	Further details
List the pre specified outcomes:	
Notes:	

Data extraction

**Outcomes relevant to your review**	**Continuous=C Dichotomous=D**	**Reported in paper (YES / NO)**
Avoidance of treatment failure i.e. avoided ICU		
Dyspnoea		
Sputum purulence/production		
FEV1		
FVC		
Quality of Life		
FOSQ		
SF36		
Nottingham health profile (part 2)		
Euroqol		
General Health Questionnaire (GHQ-28)		
SAQLI		
Other		
Adverse Events		
Readmission rate		
ED presentations		
Length of stay		
Hospital admissions		

Risk of Bias Assessment

Domain	Judgement	
**Adequate sequence generation**<br>Was the allocation sequence adequately generated?		
**Allocation Concealment**<br>Was allocation adequately concealed?		
**Blinding**<br>Was knowledge of the allocated interventions adequately prevented during the study?		
**For participants**<br>Describe the methods used to blind participants awareness of group assignment		
**For assessors**<br>Describe the methods used to blind outcome assessor(s) of group assignment for each outcome		
**Incomplete outcome data addressed**<br>Were incomplete outcome data adequately addressed?		
**Free of selective reporting**<br>Are reports of the study free of suggestion of selective outcome reporting?		
**Free of other bias**<br>Was the study apparently free of other problems that could put it at a high risk of bias?		

For Continuous data at day 0								
Outcomes	Unit of measurement	5mg group			10 mg group			Details of outcome as described in text
		n	Mean	SD	n	Mean	SD	

For Continuous data at day 0								
Outcomes	Unit of measurement	5mg group			10 mg group			Details of outcome as described in text
		n	Mean	SD	n	Mean	SD	

For Dichotomous data							
Outcome	Intervention Group			Control Group			Details of outcome as described in text
	Number with event (n)	Total number (n)	%	Number with event (n)	Total number (n)	%	

If more than one intervention please complete separate page for each intervention group

If continuous data is not presented as SD, type the format at the top of the SD column (i.e. SE, CI, p, diff)

(Adapted from: Wynne R, Botti M & Paratz J)

Comprehensive list of tools used to measure each of the above outcomes				
Outcome	Name of tool	Scale/range	Direction	Validated
**Other comments**				

(Adapted from: Wynne R, Botti M & Paratz J)

Extra notes:

-
